# Scrutinizing VTE risk factors in complex renal tumor patients: a comprehensive look at the VTE-RT-IVCTT study

**DOI:** 10.1097/JS9.0000000000000969

**Published:** 2023-12-04

**Authors:** Ru Chen, Zubing Mei, Jianhui Chen

**Affiliations:** a Department of Urology, Fujian Medical University Union Hospital, Gulou District, Fuzhou City, Fujian Province; b Department of Anorectal Surgery, Shuguang Hospital, Shanghai University of Traditional Chinese Medicine; c Anorectal Disease Institute of Shuguang Hospital, Shanghai, People’s Republic of China

*Dear Editor,*

Renal cell carcinoma (RCC) is the third most common urologic malignancy, and ~20–30% of patients present with metastatic disease at the time of diagnosis. The phenomenon of renal tumor thrombus formation in the renal vein and its propagation into the inferior vena cava is unique to RCC^1,2^.

Renal tumor with inferior vena cava tumor thrombus (RT-IVCTT) is an extremely complex condition seen in a small minority of patients with RCC^3^. When RCC surges into the inferior vena cava (IVC), it presents a significant challenge in terms of surgical management^1^. This particular scenario constitutes about 4–10% of all RCCs and escalates the surgical complexity both in terms of technical demands and potential for intraoperative and postoperative complications^4–6^. Histologically, clear cell is the most common subtype, followed by papillary, chromophobe, collecting duct, and unclassified carcinomas. The prognosis for patients with RCC varies widely and is based on a multitude of factors^7–9^. Thrombus presents a significant risk for pulmonary embolism if it becomes dislodged during surgery.

The surgical management of RT-IVCTT represents a critical condition that requires multidisciplinary coordination. The potential for catastrophic intraoperative and postoperative complications presents challenges, including bleeding, vascular injury, and pulmonary embolism. Furthermore, the occurrence of postoperative venous thromboembolism (VTE) exacerbates the complexity of the clinical decision-making process. Radical nephrectomy with thrombectomy remains the gold standard treatment^2,7,10,11^, yet significant dilemmas are faced, primarily influenced by the level of the thrombus. Surgical approach (open versus laparoscopic versus robotic), use of cardiopulmonary bypass, and routine use of renal artery embolization, all represent significant areas of debate^12–16^. Moreover, managing patients postoperatively is just as intricate. Balancing the risks and benefits of anticoagulation, particularly in those patients at high risk of VTE and hemorrhage, represents another dimension of the management dilemma. Given the rarity and complexity of RT-IVCTT, literature to guide its management is limited to retrospective case series and expert opinions, hence the importance of studies providing insights into risk factors and strategies to optimize the perioperative and postoperative management of these patients.

The recent retrospective study by Wang *et al*.^17^ represents a significant step toward deepening our understanding of VTE risk factors in patients with RT-IVCTT. We recognize that this study has contributed substantially to its significance within renal oncology and clinical oncology literature. However, some key points should be highlighted and discussed regarding the findings of this study. Firstly, while the study included patients subjected to surgical interventions, it would be relevant to clarify the specific operative approaches employed. Were patients subjected to open, laparoscopic, or robotic-assisted procedures? This is pertinent as the surgical approach could influence VTE incidence^18^. Secondly, this study shined light on the potential influence of inflammation and hematological change on VTE. Future studies may benefit from exploring molecular markers for thrombosis, such as D-dimer, cancer procoagulant or circulating tumor cells, as potential predictors of VTE^19^. Thirdly, the study did not find a significant association between neoadjuvant therapy and VTE in RT-IVCTT patients. Further interrogation regarding these neoadjuvant therapies, like their specific types and the duration for which they were administered, could contextualize the findings better. Fourthly, the study noted that patients underwent low-dose anticoagulant therapy, which was generally stopped a week prior to surgery. How would prolonged or high-dose anticoagulant use impact the occurrence of VTE in this patient population? Finally, the study found no significant relationship between smoking and VTE incidents, contradicting previous investigations^20^. A larger patient sampling focusing on smoking habits in context with RT-IVCTT might provide an illuminating perspective.

Indeed, this study also has several points of strength in the following aspects. Firstly, it is noteworthy that the authors employed a retrospective case–control design, which robustly underscored their findings. This design yields an exhaustive analysis and allows for a clear delineation of relationships between the multitude of variables and the outcome, VTE. Secondly, the contribution of a comprehensive analysis of both widely accepted and novel risk factors should not be underemphasized. Particularly, the study systematically analyzed an array of clinicopathological and hematological factors and their relationship with VTE in RT-IVCTT patients. This enhances our understanding of this complex clinical scenario. Thirdly, the most seminal point of this study is its demonstration of a significant relationship between the levels of serum albumin, neutrophils, and hemoglobin and the development of VTE symptoms. This innovative approach provides new tools for the risk stratification of these patients and might shed light on other malignancies. Finally, the study’s findings are not just interesting from a scientific perspective but have a significant clinical relevancy. The understanding of these risk factors can help craft strategies for preoperative optimization and dental management of VTE within this particular cohort, which signifies a central clinical concern in the approach to RT-IVCTT.

## Ethical approval

Not applicable.

## Sources of funding

This study is funded by grants from the Natural Science Foundation of Fujian Province (2023J011724) and the Training Program for Young and Middle-aged Elite Talents Sponsored by the Fujian Provincial Health Technology Project (2021GGA014).

## Author contribution

R.C.: conceptualization, study concept and design, and drafting of the manuscript; J.C.: drafting and validation of the manuscript; Z.M.: conceptualization and critical revision of the manuscript for important intellectual content. All authors critically reviewed and approved the final version of the manuscript before submission.

## Conflicts of interest disclosure

There are no conflicts of interest.

## Research registration unique identifying number (UIN)

1. Name of the registry: not applicable.
2. Unique identifying number or registration ID: not applicable.
3. Hyperlink to your specific registration (must be publicly accessible and will be checked): not applicable.

## Guarantors

Zubing Mei and Ru Chen.

## Data availability statement

Not applicable.

## Provenance and peer review

Commentary, internally reviewed.
